# Impaired monocyte‐derived dendritic cell phenotype in prostate cancer patients: A phenotypic comparison with healthy donors

**DOI:** 10.1002/cnr2.1996

**Published:** 2024-02-13

**Authors:** Parisa Bakhshi, Maryam Nourizadeh, Laleh Sharifi, Mohammad R. Nowroozi, Monireh Mohsenzadegan, Mohammad M. Farajollahi

**Affiliations:** ^1^ Department of Medical Biotechnology, School of Allied Medical Sciences Iran University of Medical Sciences Tehran Iran; ^2^ Immunology, Asthma and Allergy Research Institute Tehran University of Medical Sciences Tehran Iran; ^3^ Uro‐Oncology Research Center Tehran University of Medical Sciences Tehran Iran; ^4^ Department of Medical Laboratory Sciences, School of Allied Medical Sciences Iran University of Medical Sciences Tehran Iran

**Keywords:** co‐stimulatory molecules, dendritic cells, prostate cancer

## Abstract

**Background:**

Dendritic cells (DCs) play a crucial role in immunity. Research on monocyte‐derived DCs (Mo‐DCs) cancer vaccines is in progress despite limited success in clinical trials. This study focuses on Mo‐DCs generated from prostate cancer (PCA) patients, comparing them with DCs from healthy donors (HD‐DCs).

**Methods:**

Mo‐DCs were isolated from PCA patient samples, and their phenotype was compared to HD‐DCs. Key parameters included monocyte count, CD14 expression, and the levels of maturation markers (HLA‐DR, CD80, CD86) were assessed.

**Results:**

PCA samples exhibited a significantly lower monocyte count and reduced CD14 expression compared to healthy samples (p ⟨ 0.0001). Additionally, PCA‐DCs expressed significantly lower levels of maturation markers, including HLA‐DR, CD80, and CD86, when compared to HD‐DCs (*p* = 0.123, *p* = 0.884, and *p* = 0.309, respectively).

**Conclusion:**

The limited success of DC vaccines could be attributed to impaired phenotypic characteristics. These observations suggest that suboptimal characteristics of Mo‐DCs generated from cancer patient blood samples might contribute to the limited success of DC vaccines. Consequently, this study underscores the need for alternative strategies to enhance the features of Mo‐DCs for more effective cancer immunotherapies.

## 1 INTRODUCTION

Dendritic cells (DCs), as professional antigen‐presenting cells (APCs), capture, process, and present antigens and play a pivotal role in the immune system by orchestrating T‐cell immune responses. DCs also interact with other immune cells including B cells,^1^ NK cells,^2^ and macrophages.^3^ In DC biology, there are two different states of development: immature and mature. DCs differ phenotypically and functionally between immature and mature. An immature DC exhibits primitive cell characteristics, such as the expression of major histocompatibility complex class II (MHC‐II). Immature DCs act as antigen‐sampling cell, silently surveying the body for signs of invasion. Upon encountering foreign or altered self‐antigens, they undergo a remarkable transformation into mature DCs. The maturation occurs when DCs upregulate surface maturation markers such as CD80 and CD86.^4^ One essential stage in generating antitumor immunity is activation of naive T cells by mature DCs. The DC surface costimulatory molecules specifically, CD80 (B7‐1) and CD86 (B7‐2), engage CD28 and CD154, on T cell counterparts. A series of intracellular signaling processes are triggered via this complex interaction between DCs and T cells, which leads to T cell activation, proliferation, and differentiation into effector and memory cells.^5^

As cancer vaccines, DCs loaded with tumor antigens can induce antitumor activity in T cells, ultimately leading to tumor regression.^6^ Since 1995, DCs have been used in trials worldwide for a variety of indications. More than 200 clinical trials have investigated DC vaccines for various types of cancers, including melanoma, prostate cancer (PCa), glioblastoma, breast cancer, and ovarian cancer.^7^, ^8^ Sipuleucel‐T, commercially known as Provenge, stands as a pioneering milestone in the field of DC vaccines, being the first approved therapeutic of its kind. A large clinical trial, known as the IMPACT trial, found that Sipuleucel‐T could be effective in reducing the risk of death from PCa in patients.^9^

It is becoming more widely acknowledged that cancer progression induces systemic immune changes in the host. Alterations in number and function of immune cells have been identified in cancer patients' peripheral blood and lymphoid organs.^10^ The low prevalence of DC in the peripheral blood is a common technical issue in DC preparation.^11^ The immunosuppressive microenvironment in patients with PCa is orchestrated by dysfunctional DCs.^12^ Therefore, tumor‐associated DCs are defective in their differentiation and activation and are poor immune stimulators.^12^, ^13^ To address these issues, in vitro differentiated monocyte‐derived dendritic cells (Mo‐DCs) are commonly used as an alternative method for preparing DCs. In order to prepare Mo‐DCs, peripheral blood mononuclear cells (PBMC) are separated from CD14^+^ cells and cultured with cytokines, typically GM‐CSF and interleukin (IL)‐4, for 4–5 days.^14^ Mo‐DCs possess the ability to display antigens with both MHC class I and II molecules, stimulate CD4^+^ and CD8^+^ T cells, and expand antigen‐specific cytotoxic T‐cells after injection, making them an ideal candidate for generating therapeutic cancer vaccines.^15^ Mo‐DCs loaded with tumor antigen have shown safety in Phase I and Phase II clinical trials.^16^ Despite this, the Mo‐DC vaccine's efficacy continues to be suboptimal.^17^, ^18^, ^19^ Therefore, it is important to assess DCs derived from monocytes in PCa patients.

This study aims to comprehensively assess Mo‐DCs derived from PCa patients by comparing them with those generated from healthy donors (HDs). Specifically, we investigate their morphology and phenotypic markers, shedding light on potential abnormalities in the expression patterns of maturation markers. Our findings may draw attention to the hypothesis that the suboptimal efficacy of DC vaccines could be linked to low capacity of Mo‐DCs generated from patient. These insights have the potential to guide the development of more effective therapeutic strategies.

## 2 MATERIALS AND METHODS

### 2.1 Sample collection

Blood samples were collected from patients with prostate cancer and healthy donors. There were seven samples for each. Samples of HDs were collected from anonymous donors who were donating blood to the Iranian Blood Transfusion Organization (IBTO). The age and gender of the donors were specified. It is noteworthy that the healthy donors were carefully selected to match the age and gender distribution of the PCa samples. Patient blood samples were obtained from the Uro‐Oncology Research Center of the IKHC‐Tehran. The mean age of patients was 64.29 and all selected patients were diagnosed with high‐grade prostate cancer with Gleason score ≥8. Table 1 summarizes the patient characteristics, including age, Gleason scores, and serum PSA levels.

**TABLE 1:** Patient characteristics.

Patient	Age (years)	Gleason pattern/score	Serum PSA (ng/mL)
P1	76	5 + 4 = 9	867
P2	55	4 + 4 = 8	28.4
P3	65	4 + 4 = 8	143.1
P4	63	5 + 3 = 8	59.4
P5	69	4 + 5 = 9	103
P6	58	4 + 4 = 8	64.7
P7	64	5 + 4 = 9	238.8

### 2.2 Cell isolation and Mo‐DCs generation

Forty milliliters blood samples from PCa patients were collected in K3‐EDTA tubes. Using standard protocols, Mo‐DCs were generated from PBMCs. Briefly, PBMCs were isolated by utilizing Ficoll–Hypaque gradient centrifugation.^20^ Monocytes were purified from PBMCs by an indirect magnetic labeling system and using a magnetic‐activated cell separation (MACS) column (Miltenyi Biotec, USA, Cat. 130‐042‐401) following the manufacturer's instructions (Pan Monocyte Isolation Kit, Miltenyi Biotec, USA, Cat. 130‐096‐537). The numbers of collected monocytes were counted, and their viability was assessed using trypan blue staining. To obtain immature DCs, monocytes were cultured in RPMI‐1640 (Gibco, Germany, Cat. 11875101) complete medium RPMI‐1640 supplemented with 10% FBS, 100 U/mL penicillin–streptomycin (Gibco, Germany, Cat. 15070063), recombinant human GM‐CSF (100 ng/mL) (BioLegend, USA, Cat. 572916) and recombinant human IL‐4 (100 ng/mL) (BioLegend, USA, Cat. 574016) in a humidified incubator (37°C, 5% CO_2_) for 5 days. Cytokines were replenished every 3 days. The DCs were matured by adding 100 ng/mL LPS for 24 h.^21^ The same protocol was used for healthy donor samples.

### 2.3 Morphological analysis of DCs

For observing mature DCs, they were fixed on the slides using cytospin centrifuge (Thermo Scientific Cytospin 4, USA) and then stained with Giemsa dye. The morphology of DCs was then examined by optical microscope in the HD and PCa groups to determine the differences between them. The size and shape of cells were analyzed by using Fiji (ImageJ software, NIH, USA) on the microscopic pictures. More than 10 independent microscopic fields of each group were used.

### 2.4 Immunophenotyping by flow cytometry

To confirm the differentiation and maturation, cells were analyzed on Attune NxT flow cytometry (Thermo Fisher Scientific, USA) with fluorochrome‐labeled antibodies: PE mouse anti‐human CD14 (Cat. 325606), FITC mouse anti‐human HLA‐DR (Cat. 327006), PE mouse anti‐human CD86 (Cat. 374206), and FITC mouse anti‐human CD80 (Cat. 305206) all from BioLegend, USA. Monocytes, immature and mature DC cells were harvested by cell scraper and pipetting on the exact day of culture period (day 0, day 5, and day 6), centrifuged, and resuspended in ice‐cold FACS buffer (PBS containing 0.1% NaN_3_ and 2% FCS). Then, cells were aliquoted in FACS tubes and the optimized amount of conjugated mAbs was added. The tubes were incubated on ice in the dark for 45 min. Cells were washed and resuspended in the FACS buffer and immediately analyzed or fixed by the addition of 1%–2% paraformaldehyde. For isotype control, mouse immunoglobulin (mouse IgG) was used at the same concentration as other antibodies (PE mouse IgG, Cat. 400114 and FITC mouse IgG, Cat. 400101). Data were analyzed by FlowJo‐V10 software.

### 2.5 Statistical analysis

Data analysis was conducted using the SPSS statistical software package version 20 (SPSS, USA), and diagram representation was carried out using Prism™ version 8.0 software (GraphPad Inc., USA). The comparison of cellular count and expression of surface markers in PCa and HD adjusted *p*‐values were calculated by paired *t*‐test. All experiments were performed at least three times, and data were reported as mean values ± SD. *p* ≤ .05 was considered significant.

## 3 RESULTS

### 3.1 HD and PCa samples had different monocyte yields

**FIGURE 1:**
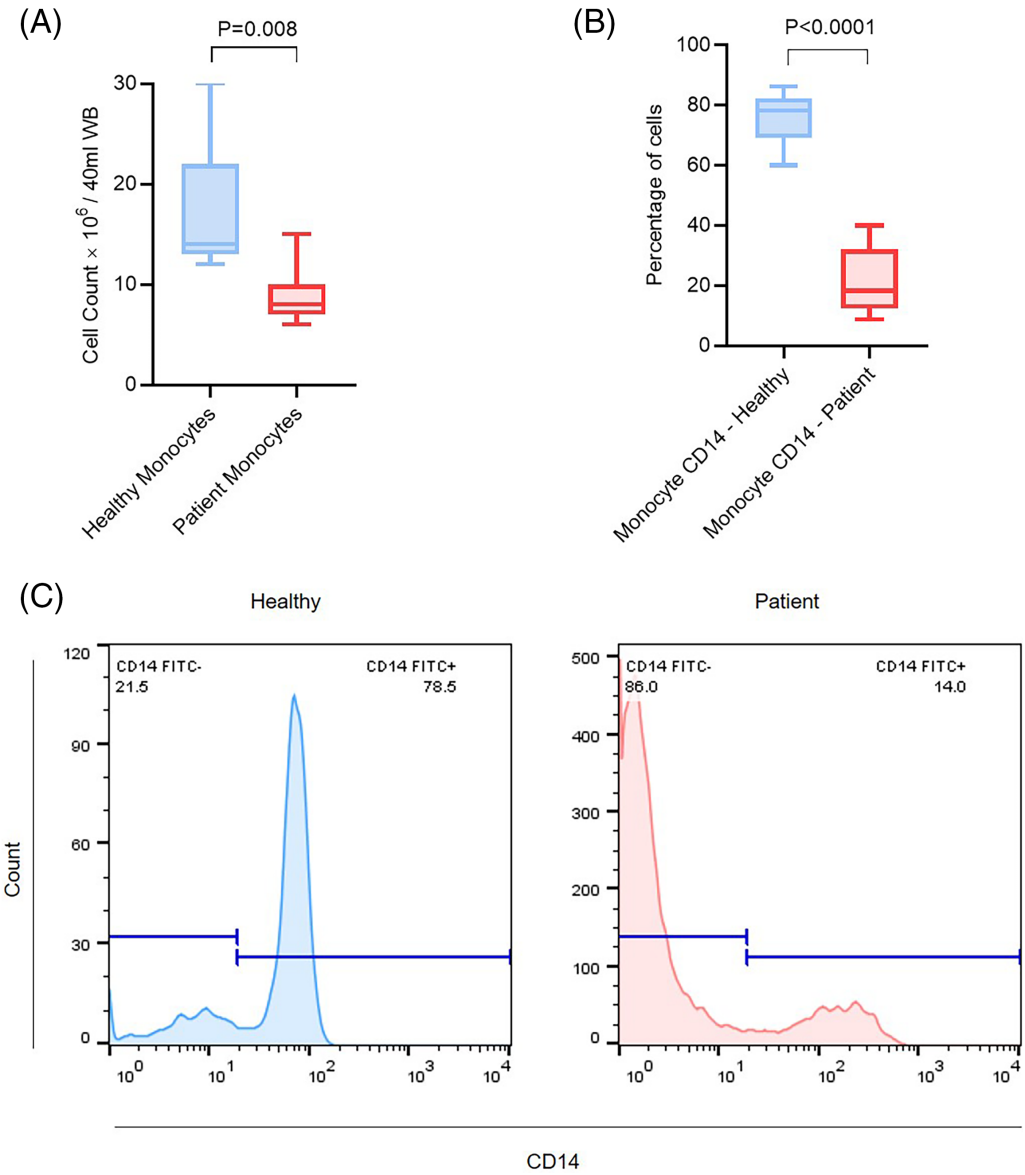
Count and flowcytometric assessment of monocytes between healthy and PCa samples. (A) Count of purified peripheral blood monocytes in healthy control (*n* = 7) and PCa patients (*n* = 7). *p* = .008. (B) Percentage of monocytes that express CD14 in healthy and PCa patients. *p* < .0001. Data are shown as box plot, with lines that indicate the mean ± SD. Paired *t* test was used to compare the difference between two groups. (C) Flow cytometric assessment for CD14 surface marker for healthy donor and patient sample respectively, gating strategy used to identify monocyte cell populations. The first gate was on physical parameters (FSC‐H vs. SSC‐H) to include all cells except debris (not shown here), and then, gating was performed in CD14^+^ and CD14^−^ cells.

Indirect monocyte separation was performed using the LS‐MACS column. The count of monocytes that were separated from 40 mL of whole blood (WB) samples was higher in the healthy samples (*n* = 7) (Mean = 17.4 × 10^6^/40 mL WB) compared to the PCa samples (*n* = 7) (Mean = 9 × 10^6^/40 mL WB) (*p* = .008, Figure 1A). The observed *p*‐value indicates a statistically significant difference, highlighting variations in monocyte counts between the two groups. Flow cytometry analysis was used to determine whether healthy and PCa monocytes expressed CD14 differently. In comparing CD14 expression levels, a paired *t*‐test was applied to evaluate whether there were significant differences between monocytes from HDs and those from PCa patients. In comparison with healthy samples, monocytes from PCa donors expressed significantly less CD14. In the PCa group, a median of 18.20 (range of 8.80%–40.00%) of the monocytes were positive for CD14, compared to a median of 78.20 (range of 60.00%–86.40%) in the HD group (*p* < .0001) (Figure 1B,C).

### 3.2 Healthy and PCa− DCs had different morphology

**FIGURE 2:**
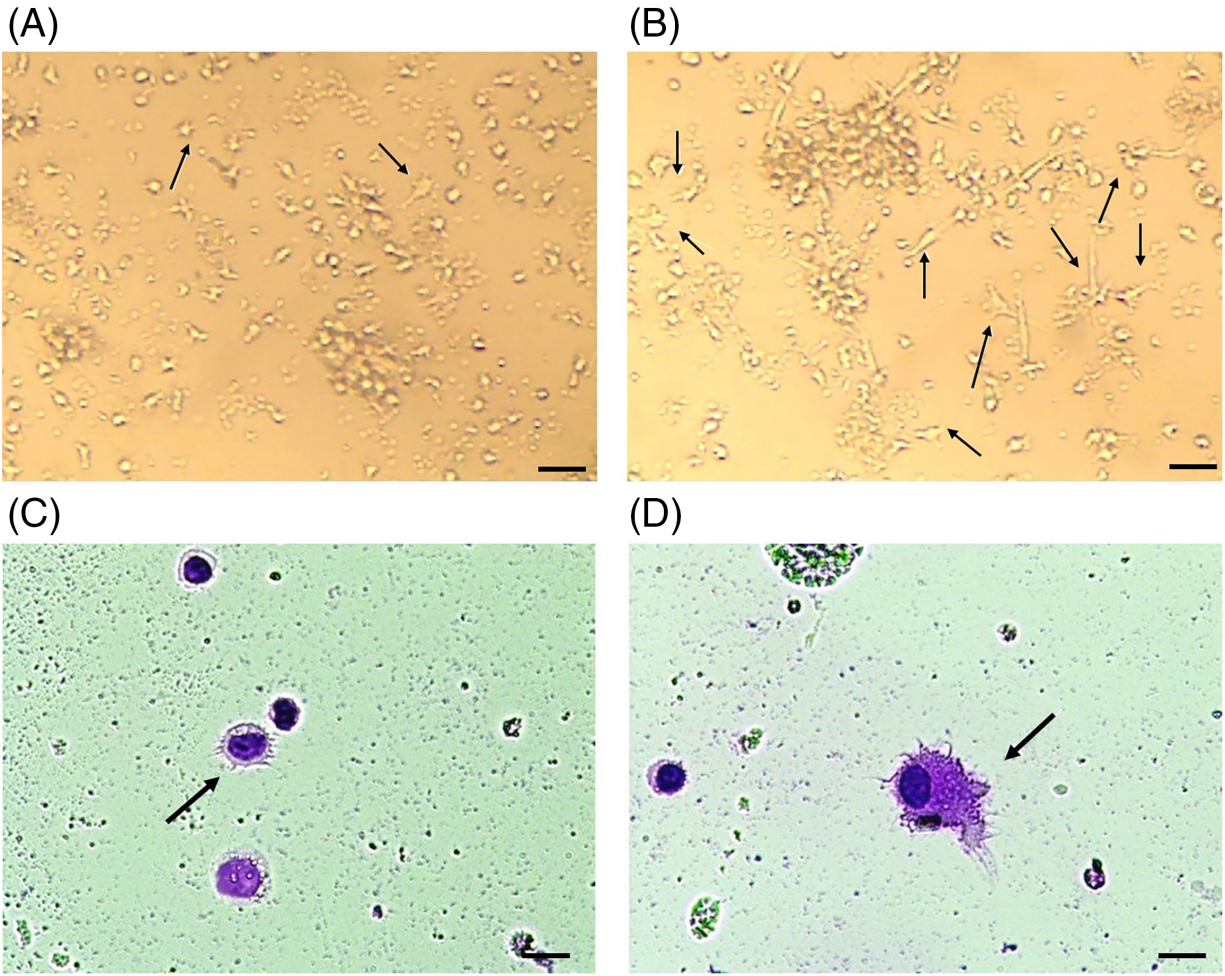
Different morphological appearance in mature DCs. (A) PCa‐DCs, (B) HD‐DCs in cell culture (white arrows), bar: 200 μm. (C) PCa‐DCs, (D) HD‐DCs stained with Giemsa Stain were observed by microscope with 40× resolution, bar: 50 μm. The morphology was assessed using Fiji (ImageJ, NIH, USA).

Observation of the mature DCs from PCa and HD samples under an invert microscope showed cells with stellate and veiled appearance (Figure 2A,B, respectively). Differential staining of cells with Giemsa stain revealed different morphologies under the microscope in both groups. As a result of LPS stimulation, mature DCs in PCa samples had circular shapes with short frills (Figure 2C). Contrary to this, Mo‐DCs in HD samples had large cytoplasm, elongated dendrites, and dense nuclear structure (Figure 2D). Approximately 47% of DCs in HD samples exhibited active appearance compared to 18% of DCs in PCa samples (*p* = .0006).

### 3.3 The expression of maturation surface markers on Mo‐DCs from PCa samples showed abnormal phenotypic characteristics

Mo‐DCs generated from patients with PCa and HD were compared for their phenotype. Immature and mature Mo‐DCs were analyzed for the expression of DC‐related surface molecules such as the MHC class II molecule (HLA‐DR), costimulatory molecules (CD80 and CD86). The expression of HLA‐DR, CD80, and CD86 in immature samples for both groups was not statistically significant. In the PCa group, a median of 31.55 (range of 26.30%–38.20%) of the immature DCs were positive for HLA‐DR, compared to a median of 54.30 (range of 32.00%–81.40%) in the HD group (*p* = .123) and for CD80 a median of 8.74 (range of 5.78%–24.30%) for PCa samples was compared to a median of 12.40 (range of 78.80%–16.20%) in the HD group (*p* = .884). A median of 49.65 (range of 12.30%–58.50%) of the immature DCs were positive for CD86, compared to a median of 52.10 (range of 35%–85.3%) in the HD group (*p* = .309).

**FIGURE 3:**
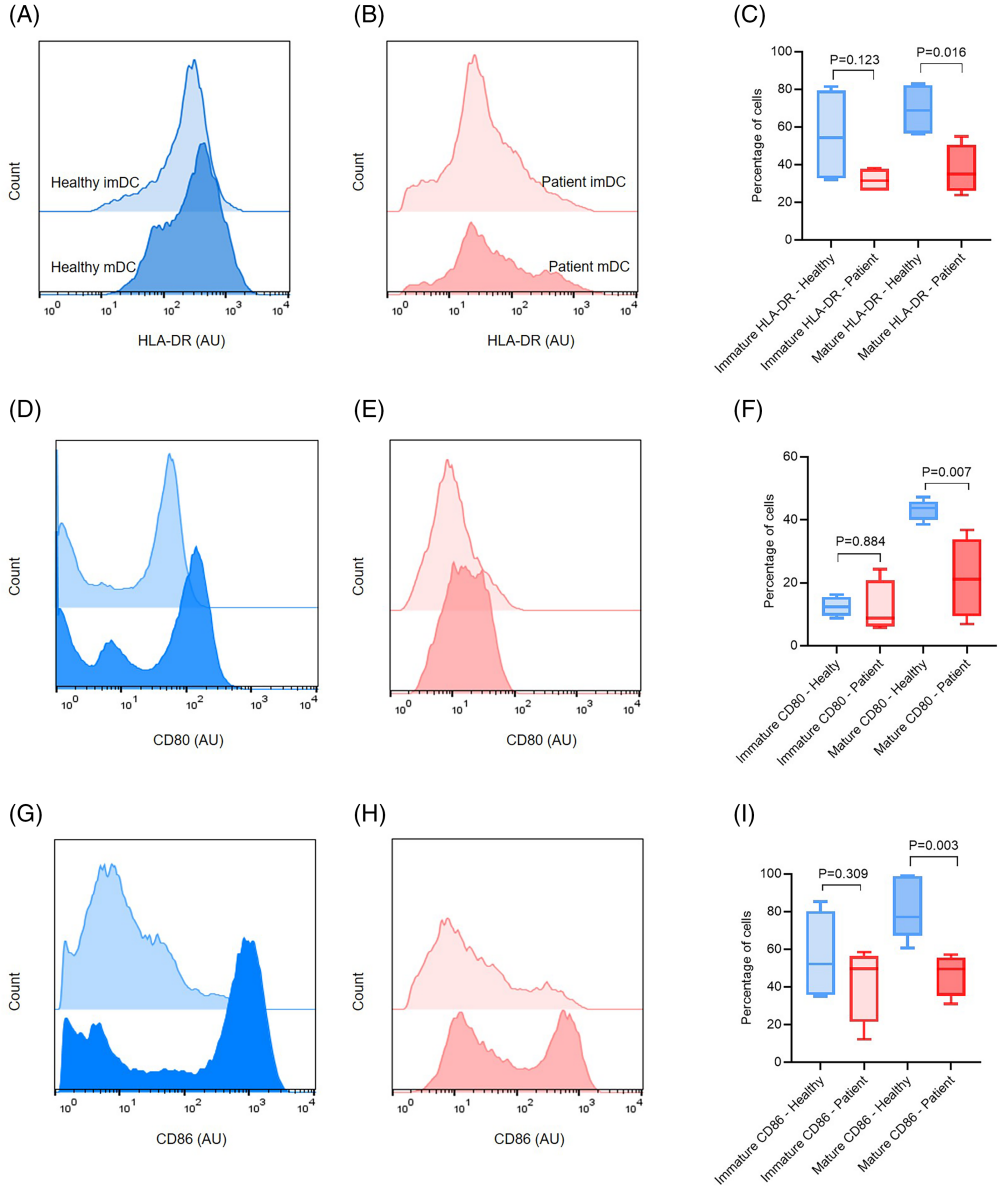
Flow cytometric assessments and quantitative analysis of the percentage of immature and mature Mo‐DCs for Co‐stimulatory markers. (A, B) HLA‐DR, (D–E) CD80, and (G, H) CD86, flow cytometric assessments for immature and mature Mo‐DCs in healthy samples (blue) (*n* = 7) and PCa samples (red) (*n* = 7). (C) HLA‐DR, (F) CD80, and (I) CD86 quantitative analysis of immature and mature Mo‐DCs between healthy and patient groups. Data are shown as box plots, with lines that indicate the mean ± SD. Paired *t*‐test was used to compare the differences between the two groups. AU, Fluorescence Intensity (Arbitrary Units); imDC, immature DCs; mDC, mature DCs.

To evaluate DC maturation, a paired *t*‐test was employed to compare the expression levels of key maturation markers (HLA‐DR, CD80, CD86) between PCa and healthy samples. After DC maturation, the expression of HLA‐DR, CD80, and CD86 in PCa‐DCs was generally lower than in HD‐DCs. The expression of HLA‐DR in healthy donors was higher than the PCa sample and statistically significant. In the PCa group, a median of 35.00 (range of 23.80%–54.90%) of the mature DCs were positive for HLA‐DR, compared to a median of 68.70 (range of 56.10%–82.90%) in the HD group (*p* = .016) (Figure 3C). Similarly, expression of the costimulatory molecules, CD80 and CD86, was significantly lower in PCa‐DCs compared to HD‐DCs (Figure 3F,I, respectively). In the PCa group, a median of 21.10 and 49.55 (range of 6.66%–36.70% and 31.00%–52.20%) of the mature DCs were positive for CD80 and CD86, compared to a median of 43.70 and 76.10 (range of 38.50%–47.20% and 60.60%–98.50%) in the HD group; *p* = .007 and *p* = .003, respectively. Collectively, these data indicate that DC maturation‐activation molecules including HLA‐DR, CD80, and CD86 phenotypically exhibited decreased expression in patients with PCa when compared to HD samples.

## 4 DISCUSSION

Our study investigated the phenotypic and morphological aspects of Mo‐DCs generated from PCa patients and HDs. We found that Mo‐DCs from PCa patients exhibited abnormal phenotypic characteristics, including reduced expression of maturation surface markers, such as HLA‐DR, CD80, and CD86. These findings suggest that Mo‐DCs from PCa patients may have impaired ability to stimulate antitumor immune responses. Several studies have shown that DCs require to express costimulatory molecules (CD86, CD83, CD80) at high levels in order to stimulate naive T cell activation and the development of memory T cells.^22^ In addition, DCs play a significant role in the immune system activation against cancers by inducing T cells for tumor‐specific cytotoxic activities.^23^ Due to the importance of the normal function of DCs in T lymphocyte activation and successful vaccination against tumors, initially, it is necessary to evaluate DCs' phenotype in terms of co‐stimulatory markers expression.

Aside from a significant difference in HLA‐DR, CD80, and CD86 expression between PCa and HD, our data revealed that the count of monocytes, as well as CD14 expression in PCa samples, were significantly lower than in healthy samples, aligning with existing literature suggesting alterations in peripheral blood cell populations in cancer.^24^, ^25^ Emerging evidences indicate that the tumor‐induced systemic environment impacts the development and phenotype of monocytes. In cancer patients, circulating monocytes exhibit phenotypic and functional alterations, including decreased expression of co‐stimulatory molecules, immunosuppressive activity, and decreased response to inflammation. Tumor‐induced reprogramming may also impair monocytes' ability to differentiate physiologically.^26^ For instance, in breast cancer patients, monocytes showed reduced expression of inhibitor of DNA binding 2 (ID2), which is vital for DC differentiation.^27^ Moreover, cancer patient‐derived DCs exhibit quantitative and functional impairments.^13^, ^28^, ^29^ Multiple tumor models have shown decreased numbers of mature DCs in the tumor bed, draining lymph nodes, and circulation.^30^

Furthermore, we found that Mo‐DCs from PCa samples had compromised morphologic features when compared to HD‐DCs. Compared to healthy mature DCs, PCa‐DCs retained circular forms with no elongated dendrites after stimulation, suggesting impaired differentiation in patients with PCa, although further research is required.

Troy et al.^31^ suggested that, DCs in PCa patients have low quantities of costimulatory molecules and are less capable of stimulating allogeneic T cell proliferation. Regarding the impaired function of circulating DCs, in vitro generation of activated Mo‐DCs can be used for ex‐vivo transferring the potent cells for fighting cancerous cells.^32^ However, there are several factors that might potentially affect the process even in vitro culture of DCs with tumor necrosis factor (TNF) or GM‐CSF and CD40 ligand (CD40L) did not enhance the expression of costimulatory molecules, suggesting that tumor cells impair DCs' differentiation.^33^

Based on our findings, the expression of maturation and costimulatory molecules such as HLA‐DR, CD80, and CD86 in Mo‐DCs from PCa samples were significantly lower than Mo‐DCs from HDs. In accordance with our data, DCs generated from breast cancer patients' monocytes had a lower capacity to induce T‐cell proliferation and triggered more regulatory T‐cells than DCs generated from healthy controls.^34^ Shinde et al.^35^ demonstrated that while the differences in expression of HLA‐DR, CD80, CD86, and other phenotypic markers between HD‐DCs and multiple myeloma (MM)‐DCs did not reach statistical significance, patients with multiple myeloma had impaired Mo‐DC functioning, which may explain why DC vaccinations are ineffective. Kvistborg et al.^36^ compared Mo‐DCs from colorectal cancer (CRC) patients and non‐small‐cell‐lung‐cancer (NSCLC) with healthy donors. As compared to DC generated from cancer patients, monocytes from healthy samples reached a significantly higher maturation stage. Compared to Mo‐DC from cancer patients, healthy Mo‐DC expressed higher levels of HLA‐DR, CD86, CD83, and CCR7.

While our study provides valuable insights into the phenotypic and morphological characteristics of Mo‐DCs in PCa patients, it is essential to acknowledge certain limitations. First, our sample size, although carefully selected, is relatively small, and this may impact the generalizability of our findings. Additionally, the lack of functional assessments limits our ability to draw definitive conclusions about the functional implications of the observed phenotypic differences. Furthermore, our study primarily focused on in vitro‐generated Mo‐DCs, and extending these findings to the in vivo setting requires cautious consideration of the complex tumor microenvironment and systemic influences. Addressing these limitations will be crucial in guiding future research directions in this field. Despite the limitations, our study sheds light on the altered phenotype and impaired maturation of Mo‐DCs in PCa patients. Understanding these phenotypic distinctions may have implications for the development of immunotherapeutic strategies. The compromised maturation and reduced expression of co‐stimulatory markers in Mo‐DCs from PCa patients suggest potential challenges in utilizing these cells as cancer vaccine. Recognizing these obstacles prompts further investigations into refining protocols for Mo‐DC generation.

The clinical significance lies in the prospect of enhancing the efficacy of DC‐based immunotherapies by addressing the observed phenotypic alterations. Our findings have important implications for the development of DC‐based cancer vaccines. Since Mo‐DCs from PCa patients are likely to have impaired ability to induce antitumor immune responses, it may be necessary to develop new methods for generating Mo‐DCs from PCa patients that are more effective. For instance, we conducted an additional experiment in which PCa Mo‐DCs were pulsed with a long peptide, and the maturation period was extended from 24 to 48 h to ensure enhanced maturation and functionality. The results of the functional study indicate that PCa Mo‐DCs exhibit the capability to activate T cells and induce tumor cell death.^37^ Additionally, it may be necessary to use combined DC‐based vaccines with other cancer treatments to improve their efficacy.^38^, ^39^ Future studies with larger cohorts and functional assessments will be crucial for validating and extending our findings, ultimately paving the way for more effective DC‐based cancer vaccines. For example, the incorporation of real‐world clinical‐genomic data from initiatives like the Prostate Cancer Precision Medicine Multi‐Institutional Collaborative Effort (PROMISE)^40^ and insights from diverse immunotherapeutic approaches in prostate cancer, including microbial‐based immunotherapy,^41^ could synergistically complement our findings. This collaborative approach would contribute to a more comprehensive understanding of the dynamics influencing PCa immunotherapy.

## 5 CONCLUSION

To sum up, we propose a phenotypic feature of DCs made from PCa patients' monocytes differs from healthy Mo‐DCs. Using the same conditions for generating DCs from monocytes, Mo‐DC from HDs were found to display mature phenotypic characteristics, in contrast to Mo‐DC obtained from PCa samples. Although the molecular characteristics of Mo‐DC from PCa patients are lower than those from HDs, which may affect their function, there are still different approaches to making Mo‐DCs useful as adjuvants in cell‐based cancer vaccines. Importantly, these observed differences underscore the clinical significance of understanding the altered phenotype of Mo‐DCs in PCa. Future investigations exploring the molecular intricacies behind these phenotypic variations could unravel novel therapeutic targets, paving the way for more effective DC‐based cancer vaccines.

## AUTHOR CONTRIBUTIONS

**Parisa Bakhshi:** Data curation (equal); formal analysis (equal); investigation (equal); methodology (equal); resources (equal); software (equal); writing – original draft (equal); writing – review and editing (equal). **Maryam Nourizadeh:** Data curation (equal); investigation (equal); project administration (equal); visualization (equal). **Laleh Sharifi:** Preparing samples. **Mohammad. R Nowroozi:** Preparing samples. **Monireh Mohsenzadegan:** Conceptualization (equal); data curation (equal); investigation (equal); project administration (equal); supervision (equal); writing – review and editing (equal). **Mohammad M. Farajollahi:** Funding acquisition (equal); investigation (equal); supervision (equal); writing – review and editing (equal).

## CONFLICT OF INTEREST STATEMENT

The authors have stated explicitly that there are no conflicts of interest in connection with this article.

## ETHICS STATEMENT

Peripheral blood collection, processing, and all experimental procedures were approved by the Ethics Committee of the Iran University of Medical Sciences (IR. IUMS.1398.090). All participants provided fully informed consent before the blood samples were collected.

## Data Availability

Data sharing is not applicable to this article as no new data were created or analyzed in this study.
